# Supplementary material for: Are carbon emissions trading and green financial instruments synergistic? -Comprehensive quantitative research based on content analysis
Source: PLoS One. 2024 Mar 7;19(3):e0298601. doi: 10.1371/journal.pone.0298601 (PMC10919601; doi:10.1371/journal.pone.0298601)
Supplement: S1 Data — (ZIP) [file pone.0298601.s001.zip › Data/地级市Z_F工作报告.zip!/║■▒▒/╧╠─■╩╨/└┤╫╘╣1⁄2╓┌║┼í╛┬φ┐╦╩2╛▌═°í┐_2014.docx]

各位代表：

现在，我代表市人民政府向大会报告工作，请予审议，并请市政协委员和列席人员提出意见。

一、2013年工作回顾

2013年，在省委、省政府和市委的坚强领导下，在市人大、市政协和社会各界的监督支持下，市政府紧紧依靠全市人民，牢牢把握“去稳竞进”的总基调和“两个满分”的总要求，解放思想，攻坚克难，省级战略咸宁实施取得重要进展，市四届人大三次会议确定的目标任务基本完成。

综合实力再攀新高。初步测算，全市生产总值872.11亿元，增长10.6%，增加额连续4年超过100亿元；全社会固定资产投资971.84亿元，增长28.5%；社会消费品零售总额320.64亿元，增长13.8%；公共财政预算收入58.71亿元，增长28.1%。中国恒天、华中绿色印刷包装、百翔电池、津晶城科技园、台湾DTE消防液、大润发物流、雨润物流等项目成功落户，南玻华中产业园建成投产，华润（赤壁）电厂3号、4号机组并网发电。新引进项目和续建项目1201个，到位资金580.1亿元，增长85.9%，新签约、开工和投产项目均创历年之最。

产业转型步伐加快。全市三次产业结构调整为18.7：48.7：32.6。工业崛起逆势奋进。全市规模以上工业增加值430.17亿元，增长13.6%；净增规模以上工业企业106家，总数达到780家。崇阳钒产业新进入全省重点成长型产业集群，“一县一集群”格局形成。高新技术企业41家，其中新增19家；高新技术产业增加值46.61亿元，增长16.8%。特色农业速效并进。粮食生产实现“十年增”。建成农业产业化板块基地350万亩，省级重点龙头企业44家，其中新增19家。启动实施竹、茶、油茶三个百亿元产业工程，咸安竹产业园、赤壁砖茶产业园、通山厚生祥红茶文化园、黄袍山国家油茶产业示范园建设加快推进，全市油茶面积发展到42万亩，全国油茶现场会在通城召开。现代服务业开拓求进。咸安商贸物流园、嘉鱼潘家湾物流聚集园、赤壁沃尔玛购物广场、通城泽中物流园、崇阳百盟一马商贸物流产业园、通山石材物流园等项目加快推进，中国首个绿色生态产业展览交易基地落户赤壁。接待游客2650万人次，旅游总收入130亿元，分别增长26%、20 .8%。商品房竣工面积170.45万平方米，销售面积271.31万平方米。

创新驱动成效明显。“两型”社会建设综改试验深入推进，“五个一体化”进程加快。市区城市管理体制进一步理顺。食品药品监督管理局、卫生和计划生育委员会顺利组建。预算体系建设全省领先，市区（咸宁经济开发区、咸安区）财税体制调整完善，“营改增”试点稳步推进，市直公务卡管理全覆盖。金融创新取得较大突破，以全省第1名的成绩获评全省金融信用市。通山、崇阳、咸宁农村商业银行相继组建，“政银保”模式创全国先河，通山县域金融工程试点成为全省样板，全市16家企业登陆武汉股权托管交易中心挂牌。城乡建设用地增减挂钩、低丘缓坡土地综合利用试点扎实推进，争取部、省国土整治项目29.5万亩。开放合作谱写新篇章。实际利用外资2.47亿美元，增长16.7%；出口2.91亿美元，增长25.3%。与岳阳市、九江市、安庆市签署《咸宁共识》，打造长江中游城市群先行区、示范区；与韩国义王市、匈牙利威莱士艾及哈兹市缔结友好城市。全市各类市场主体15.34万户，增长21.37%；新登记市场主体2.14万户，增长16.02%。

城市品质持续提升。在全省率先开展绿色崛起规划和全域新型城镇化总体规划编制，智慧（数字）城市地理信息框架系统建成使用。“六城三区”建设力度加大，成为全市最具潜力、最富活力的板块。与中城建开元（北京）公司开展战略合作，湿地生态新城呼之欲出。武咸城际铁路建成开通，咸宁率先进入高铁、城铁、普铁的“三铁”时代。中心城区外环高速初具雏形，淦河下游综合治理二期、南外环人工湖二期、十六潭公园三期及十六潭路改造、城际铁路垂直绿化、一号桥泵站迁改等项目建成使用，咸宁旅游集散中心、潜山古寺即将向市民开放，香城大道和泉都大道改造升级、百盟温泉欢乐世界、香泉映月旅游度假区、中商购物广场、天成商业综合体、城铁南站宏大国贸广场，以及传媒大厦、妇儿活动中心、科技馆、咸宁文化城等项目加快推进。创建国家森林城市、国家环保模范城市、国家双拥模范城市工作扎实推进，创建国家卫生城市工作通过评估，成功摘取全国绿化模范城市、国家园林城市两项“国字号”荣誉。第五届国际温泉文化旅游节成功举办，旅游小姐菁英赛、百强旅行社牵手咸宁活动叫响全国，“香城泉都”品牌越擦越亮。

城乡统筹扎实推进。城乡规划体系逐步完善，城乡面貌不断改善。咸通高速、咸黄高速竣工通车，武深高速嘉通段、武汉城市圈环线高速咸宁西段、幕阜山生态旅游公路、武咸快速通道、咸潘一级公路等项目加快推进。高速公路通车里程313公里，全市交通投资50.19亿元，再创历史新高。嘉鱼县潘家湾镇列入全省“四化同步”试点镇，赤壁市赵李桥镇羊楼洞村列入全国首批“传统村落保护名录”，新增10个省级新农村建设示范村，12个村庄被命名为全省“宜居村庄”。美丽村庄创建活动深入开展，村企合作共建模式逐步推广。“三万”活动取得阶段性成效，农村垃圾清运体系初步形成。幕阜山片区扶贫攻坚全面启动，全市累计减贫61023人。生态文明建设力度加大，完成造林30.5万亩，垃圾焚烧发电项目顺利建成，赤壁凯迪生物质发电项目试运行，我市被确定为全国水生态文明建设试点城市，省定节能减排任务圆满完成。

民生事业全面进步。城镇居民人均可支配收入18581元，增长9.9%；农村居民人均纯收入8480元，增长13%。义务教育均衡发展稳步推进，赤壁市通过教育部义务教育均衡发展评估。咸宁实验外国语学校动工兴建，实验小学、温泉中学改造升级，高等教育、职业教育创新发展。公共文化服务体系建设加强，新增国家重点文物保护单位6处，涌现出一批优秀精神文化产品。社会科学、文学艺术、新闻出版、广播影视事业发展加快。医药卫生体制改革深入推进，在全省率先启动城镇居民大病保险，村卫生室标准化建设达标率100%，公共卫生保障能力提升。低生育水平稳定，出生人口性别比降低。全民健身运动广泛开展。全市新增城镇就业4.73万人，城镇登记失业率2.88%；五项社会保险参保163万人次，新型城乡居民养老保险参保104万人，参保率98%；新农合参合208.32万人，参合率99.77%，住院费用补偿比76.1%。城乡低保惠及12.67万户、20.51万人。新开工建设保障性住房6526套，发放廉租住房补贴1365户；实施农村危房改造7039户，竣工率100%。社会治理创新取得新成效，信访维稳工作得到加强，易肇事肇祸精神病人集中救治和吸毒人员强制收戒经验在全省推广。“平安咸宁”建设扎实推进，打击“一霸四强”力度加大，安全生产和食品药品安全形势保持稳定，全年无重大安全事故发生。除老年人活动中心跨年建设外，市政府年初承诺的10件实事圆满完成。

国防动员和后备力量建设进一步加强。对口支援新疆、建始县工作深入推进。妇女儿童、青少年、关心下一代、老龄、残疾人等事业全面发展。对台、侨务、外事、科普、保密、档案、方志、统计、气象、人防、民族宗教、防震减灾等工作取得新的成绩。县（市、区）再夺一批“国字号”。咸安、通城、赤壁荣获全国科技进步先进县（市、区）；崇阳荣获全国第二届国土资源节约集约模范县、全国法治县创建先进单位；嘉鱼荣获全国平安建设先进县；通山荣获全国群众体育健身先进县；赤壁入选第二批全国旅游标准化试点市；咸安获批国家知识产权强县工程试点区。

过去的一年，我们自觉在市委的领导下履行职责，自觉接受人大法律监督、工作监督和政协民主监督，办理人大代表建议70件、政协委员提案140件，办结率100%，满意率不断提升。认真贯彻中央八项规定、省委六条意见和市委七项要求，落实“三短一简”，推进治庸问责，深入开展转变作风优化环境集中整治活动，积极参与党的群众路线教育实践“三同步”活动，带头密切联系群众，带头解决实际问题，政府机关作风进一步转变。完善政府工作规则，开展市政府集体学习活动，下放和取消审批权限177项，启动市政务服务中心“二次创新”，政务服务水平进一步提升。加强惩治和预防腐败体系建设，政府系统党风廉政建设责任制得到有效落实。

回顾过去的一年，困难比预想的多，成绩比预料的好。面对错综复杂的形势和严重干旱等自然灾害，我市经济社会能够保持持续健康发展，实属来之不易。这是省委、省政府和市委坚强领导、科学决策的结果，是市人大、市政协和社会各界有效监督、全力支持的结果，是全市人民齐心协力、顽强拼搏的结果。在此，我代表市人民政府，向全市广大干部群众，驻咸部队、武警官兵和公安干警，各民主党派、工商联、无党派人士，以及所有关心支持我市发展的各界朋友，表示衷心的感谢并致以崇高的敬意！

在肯定成绩的同时，我们也清醒地看到存在的问题，主要是：经济总量不大，产业支撑力不强，城镇化水平不高，环保压力较大，市场机制发挥不充分，公共服务和保障水平较低，少数部门和干部作风不实、落实不力等。对此，我们将加大改革创新的力度，切实采取有效措施加以解决。

二、2014年工作安排

2014年是贯彻落实党的十八届三中全会精神、全面深化改革的开局之年，是全面建成小康社会决定性阶段的重要一年，也是加快推进省级战略咸宁实施的关键之年。综观大势，我们仍然处于可以大有作为的黄金发展期。一方面，世界经济转好趋势增强，欧美发达国家加大了技术创新力度，新一轮科技革命正在孕育之中；我国进入打造经济升级版新阶段，党的十八届三中全会作出全面深化改革的历史性决定，开启了新一轮“改革红利”的美好前景。另一方面，中央对湖北“建成支点、走在前列”的新定位，以及长江中游城市群建设、省委“两圈两带”战略实施和幕阜山片区扶贫攻坚等机遇，坚定了我们加快发展的信心和决心，“三铁一带”拉近了我市与大城市的时空距离，区域联系和经济要素流动速度加快。同时，我市良好的生态环境、积蓄的发展后劲、凝聚的发展共识，必将为我们带来更多正能量。只要我们切实增强进取意识、机遇意识、责任意识，坚持“拼”字当头，不松气、不泄气、憋足一口气，就一定能够开创省级战略咸宁实施的新局面。

今年政府工作的总体思路是：认真贯彻落实党的十八大、十八届三中全会、省委十届三次四次全会和市委四届三次全会精神，坚持“速效兼取、去稳竞进”的工作总基调，牢牢把握市场、绿色、民生“三维”纲要，把改革创新贯穿于经济社会发展各个领域各个环节，着力发挥市场的决定性作用，着力转变经济发展方式，着力保障和改善民生，着力提升政府执行力、服务力，奋力推进省级战略咸宁实施干在实处、走在前列。

全市经济社会发展主要预期目标是：生产总值990亿元，力争突破1000亿元；规模以上工业增加值480亿元；固定资产投资1100亿元；社会消费品零售总额365亿元；公共财政预算收入68亿元；城镇居民人均可支配收入20800元；农村居民人均纯收入9500元；居民消费价格涨幅控制在4%左右；城镇登记失业率控制在4.6%以内；人口自然增长率控制在6.5‰以内；确保完成省定节能减排任务。

当前，我市面临着加快发展、加快转型，既要做大总量、又要提升质量和竞争力的多重任务。机遇稍纵即逝，挑战愈加严峻。“去稳竞进”，是时代赋予当代咸宁人的光荣职责。我们要去思想保守之稳，在改革创新上竞进；去固步自封之稳，在加快发展上竞进；去甘于平庸之稳，在比学赶超上竞进。我们要的发展是更新理念、更高水平、更优品质的发展，是环境更优美、城乡更宜居、百姓更幸福的发展，是人民群众看得见、摸得着、享受得到的发展。为此，我们要在现有基础和良好态势上，树立更加积极的目标，保持更加竞进的状态，拿出更加拼搏的作为，交上更加精彩的答卷！

围绕上述目标任务，重点做好以下五个方面的工作：

（一）坚持转方式、优结构，推动经济提速提质

加快推进工业崛起。围绕做大总量、优化结构、提升层次，实施“一工程三计划”，使鄂南强市大厦建立在稳固的工业基础之上。实施“大产业、大企业、大园区”工程，加快推进德力林浆纸一体化、嘉麟杰纺织、华琪生物、纳米轮胎，以及广东工业园、小榄工业园、咸安桑德环保产业园、嘉鱼汉麻产业园、华润（赤壁）电厂循环经济产业园、崇阳生物医药产业园、通山大盘垅工业园等项目和产业园区建设，确保绿孚生物、宇顺电子、三三重工、金海达薄瓷板、新忆通机车、兴民钢圈、玉立樊牌等项目建成投产，打造我市工业新团队。实施传统产业提升计划，推动咸安机电、嘉鱼管材、赤壁应急安全等传统优势产业升级增效。实施新兴产业培育计划，重点培育和发展先进装备制造、电子信息、生物医药、新材料、新能源汽车、节能环保等战略性新兴产业，大力推进人福新药、蒲瑞科新材料、瀛通电子数据线等产品开发，推动奔驰方向机、美国驰派达、合加环境专用汽车等汽车零部件产业加快发展。实施资源性产业开发计划，支持各地依托资源禀赋发展特色产业，加快推进通城陶瓷、崇阳绿色钒产业、通山石材产业集群发展，延长产业链，提高产品附加值。加快企业上市步伐，力争新增100家以上规模以上工业企业，新增1—2个中国驰名商标。进一步做好咸宁核电、震纶纺织城等项目前期工作。推进咸宁经济开发区创建国家级开发区、高新区。推动信息化与工业化深度融合，推广云技术运用，培育一批“两化融合”示范企业。完善政策扶持、要素保障、考核激励等机制，夯实实体经济大底盘。

加快发展现代农业。以“农业强、农民富、农村美”为目标，加快传统农业向现代农业跨越。稳定粮食生产。实施优势特色农产品区域规模化布局，推进县市区建十万亩、乡镇建万亩、村建千亩农业产业化基地，形成一批“粮油果蔬茶畜”专业大县、专业大镇。围绕打造百亿元竹、茶、油茶产业，建设国家楠竹生产农业标准化示范区，打造百公里茶叶走廊，创建国家油茶示范市。加快建设农业新技术示范基地、科技推广基地和新型农民培训基地，提高农业科技服务水平。突出抓好农产品精深加工，加快农产品加工园区和品牌建设，推进农业全产业链发展。加快构建新型农业经营体系，创新农村金融产品，搞活农村产权市场，发展多种形式的规模经营。加大农业产业化龙头企业、农民专业合作社、农业专业大户、家庭农场等农业主体培育力度，培育现代农业的“领头雁”。加强农业标准化建设，新增“三品”认证30个、国家地理标志认证1—2个。强化农产品质量安全监管，确保“舌尖上的安全”。

加快发展现代服务业。以区域性金融中心、物流中心、旅游中心建设为重点，促进服务业发展提速、比重提高、水平提升。积极引进区域外金融机构在我市设立地区总部和后台服务中心，推动设立地方法人保险、信托、金融租赁、互联网金融、期货交易机构，支持设立金融控股公司，发展小额贷款公司、担保公司。继续推进商贸企业成长工程，大力发展批发市场、配送中心、仓储运输、信息服务等物流服务网络，引进大型商贸企业和电商企业落户我市，形成区域性商贸物流中心。积极引进服务外包产业总部基地，打造中国版的“班加罗尔”。围绕建设旅游经济强市，加快推进温泉生态旅游新城、赤壁三国文化旅游试验区、通山九宫山生态旅游区、咸安桂竹生态旅游区、崇阳商周生态农业旅游区、嘉鱼江南水乡旅游区、通城瑶族原生态旅游区等“一城六区”建设。进一步整合旅游资源，丰富完善旅游产品，大力发展休闲娱乐业，推进智慧旅游项目建设，巩固旅游标准化成果，力争5A景区创建取得突破性进展，全年接待游客3200万人次以上，打造全国优秀旅游目的地。实施文化产业发展工程，打造文化产业聚集区，建设国家级绿色印刷包装物流产业基地。积极支持4G网络建设应用，大力发展物联网、云计算等新兴产业。

扩大投资和消费需求。坚定不移地抓项目、扩投入，促进投资稳定增长和结构优化。加强重大项目谋划，完善万亿元项目库，争取更多项目列入国家和省级重点项目建设计划。突出抓好交通枢纽项目建设。继续做好岳咸九铁路、咸宜吉铁路、洪崇高速公路等项目前期工作，加快推进武深高速、武汉城市圈环线高速、通界高速、武咸快速通道、幕阜山生态旅游公路等交通路网建设。确保咸潘一级公路建成通车。力争咸宁（嘉鱼）长江公路大桥、赤壁长江公路大桥开工建设，早日迎来“大桥”时代。创新投资方式，鼓励民间投资，提高社会投资比重。把扩大居民消费作为扩大内需的主要着力点，加快培育和扩大电子商务、网络文化、数字家庭、智能家电等热点消费，会展、旅游、社区、健康、信息、文化创意等服务消费，促进住房、汽车消费和养老产业健康发展。深入实施“万村千乡”市场工程和“新网工程”，推动便利消费进农村、进社区。

（二）坚持创新驱动，全面深化改革开放

大力推进科技创新。全面深化科技体制改革，加大科技投入，争创全国科技进步先进市，做实省科技成果转化示范基地。实施高新技术产业发展工程，力争新增认定高新技术企业20家以上，高新技术产业增加值占GDP比重提高到7%以上；加快高新技术产业园区建设，支持各类开发区、工业园区建设高新技术产业孵化园。推动建立产学研相结合的协同创新体系，构建一批面向企业特别是中小企业的技术创新服务平台，引导创新要素向企业聚集。加强知识产权保护。创新人才发展机制，完善人才政策和服务体系，加大引进科技人才和企业经营管理人才力度。把咸宁经济开发区建成人才特区、创新发展特区。

着力激发创新活力。处理好政府和市场的关系，使市场在资源配置中发挥决定性作用。牢固树立“产业第一、企业家老大”的理念，加大简政放权力度，探索试行清单管理模式，深入推进行政许可项目清理，加快市政务服务中心“二次创新”，建成全省政务服务最优的市州。整合政府经营性资产，完善国有资产监管体制，加强与央企、省企对接合作。放宽市场准入，繁荣发展民营经济，推动民间资本进入基础设施、基础产业和民生事业等领域，鼓励全民创富。加快发展和积极利用多层次资本市场，提高直接融资比重。支持发展创业投资、股权投资，支持企业发债融资。稳步实施市域金融工程，壮大县域金融板块。健全财政预算体系，大力推进预算支出绩效管理，稳步推进预算信息和“三公”经费公开。加强政府债务管理，管好用好重大产业发展基金，完善城投、荣盛投资、交通投资、职教投资、旅游投资等投融资平台运行机制。提高住房公积金使用效益。继续抓好城乡建设用地增减挂钩、低丘缓坡土地综合利用试点，推进节约集约用地。积极稳妥推进机构人事制度改革、事业单位分类改革。鼓励各地先行先试、大胆创新。

扎实推动开放合作。抢抓国家扩大内陆开放新机遇，以开放促改革促发展。落实《咸宁共识》，积极推进长江中游城市群区域合作。加快推进武汉新港咸宁港区建设，大力发展沿江经济、临港经济，支持咸嘉新城发展飞地经济。继续做好海关、商检机构设立工作，加强与东湖综合保税区对接合作，探索建立双边贸易区。把招商引资作为扩大开放的主攻方向，完善招商机制，创新招商方式，大力开展产业链招商、以商招商，重点瞄准世界500强、国内500强企业招商引资，进一步做好恒天、华帝、海尔等企业跟踪对接工作，提高招商引资的质量和效益。加强出口基地建设，重点培育机电、纺织、建材、医用卫材等优势出口产业，推动出口基地升级为国家级、省级专业型示范基地。加强出口主体培育，继续做好开口不出口和异地出口企业的转化工作。扩大对外劳务合作，大力引进海外高层次人才，加强国际友好往来，多渠道扩大对外开放。

（三）坚持“四化”同步，统筹推进城乡协调发展

实施城市提质工程。围绕打造“香城泉都”，提升城市品质，建设秀美、浪漫、和谐的魅力城市。向东，重点抓好香城大道、泉都大道改造升级和东外环、咸宁大道延伸等工程建设；向南，重点抓好马柏路综合整治，重拳打击“三乱”、“两违”，畅通城市出口；向西，重点抓好淦河大桥、龙潭大桥修建和南站路、西外环等工程建设，打通城际铁路与咸宁大道的连接；向北，重点抓好107国道咸安绕城段和高铁新区、站中大道等工程建设，抓好鱼水路、站前广场综合整治，提升城市窗口形象。在主城区，着力抓好交通整治，改善交通循环；加快城际铁路沿线开发；加大背街小巷、“城中村”和棚户区改造力度。在“六城三区”，按照“产城一体、园城一体、港城一体”的思路，加快道路、供水、供电、环保等基础设施建设，加大板块招商力度，实现组团式发展。提升城市文化功能，重修永安阁、三元书院，启动建设茶博馆，基本完成潜山古寺及配套工程，彰显城市文脉。提升城市综合服务功能，调整优化公交线路，增加公交港湾、首末站，完善休闲、健身、公厕、停车泊位等服务设施；发展特色餐饮，打造夜市、特色街和城市商业综合体；建设中央商务区，打造咸宁金融港。提升城市生态功能，突出显山露水见园林的城市特色，保护山体水体，抓好城市绿化，加强环境监测，进一步优化环境质量。深入推进国家卫生城市、国家森林城市、国家环保模范城市、国家双拥模范城市等“多城同创”，继续办好国际温泉文化旅游节，扩大城市知名度、美誉度，提升市民自豪感、幸福感。

实施城乡共建工程。坚持规划引领、产业支撑，加快全域新型城镇化总体规划编制实施，推进“六规合一”、“五区共进”。进一步抓好县市城区建设，推动县市城区合理规划、彰显特色、完善功能。积极推进新型城镇化，统筹抓好城乡一体化试点，稳步推进经济发达镇行政管理体制改革和强镇扩权改革，加快推进横沟桥镇、赤壁镇等中心镇、特色镇建设，高标准推进嘉鱼潘家湾镇省级“四化同步”示范试点建设。加强城乡规划管理，加大城乡主干道“三乱”整治力度，严格规范农村建房。以新农村建设为抓手，大力推进宜居村庄建设，进一步抓好美丽村庄创建活动，弘扬耕读文明。结合“万名干部进万村惠万民”活动，加强农村道路、安全饮水、农田水利等农村基础设施建设，改善农村生产生活条件。切实推进农村网格化建设，解决农村公共服务“最后一公里”问题。

实施市域一体工程。积极研究咸宁与武汉的关系，推进武咸同城发展。做大做强市区经济，力争咸宁经济开发区实现固定资产投资100亿元以上、规模以上工业增加值30亿元以上、税收10亿元以上，有150个以上项目签约、开工、投产。加快发展县域经济，支持咸安、赤壁、嘉鱼率先发展，支持崇阳、通城、通山争先进位。鼓励有条件的乡镇抓好特色园区建设，壮大镇域经济。加快推进幕阜山片区区域发展与扶贫攻坚，完成60个村整村推进任务，实现片区减贫6万人。

（四）坚持绿色引领，建设生态文明示范区

加大生态文明创建力度。加快编制《咸宁绿色崛起发展规划》，深入推进“两型”社会建设，打造国家可再生能源应用示范城市，推进全国水生态文明城市试点、全国碳排放交易试点，积极创建全国生态文明先行示范区。支持咸宁经济开发区创建国家级低碳示范园区，支持赤壁建设中国绿色生态产业展览交易基地，支持崇阳建设国家农产品主产区，支持通城、通山创建国家生态功能区试点，支持咸安创建全省林业生态示范县。加强生态文明制度建设，增强全民节约意识、环保意识、生态意识。

加大生态建设和环境保护力度。深入推进天然林保护、退耕还林、沿江（库）防护林等重点生态工程，确保完成30万亩造林任务。突出抓好淦河、陆水河和幕阜山等重点水域流域生态保护和综合治理，促进生态脆弱地区生态恢复。支持通山富水湖申报国家湿地公园，支持赤壁葛仙山申报省级自然保护区。加强饮用水源地保护、建设施工废气与噪声超标治理。以创建生态镇、村为抓手，加强农业面源污染治理，建立农村垃圾处理长效机制。实施空气质量监测制度，制定雾霾应急处置预案，让咸宁天更蓝、地更绿、水更净。

强力推进节能减排。落实最严格的耕地保护和水资源管理制度。强化重点企业、行业节能减排责任，引导和支持企业加强节能技改，调整产品结构，降低能耗和生产成本。严格控制高耗能、高排放和产能过剩行业新上项目。加强城镇和工业园区污水、垃圾处理设施建设和运行监管。推广应用先进节能和环保技术产品。推进企业清洁生产，发展循环经济。

（五）坚持底线思维，让人民过上更好生活

加快发展民生事业。深化教育领域综合改革，大力促进教育公平。深入推进义务教育均衡发展，启动实施新一轮学前教育三年行动计划，提升高中教育质量，加快现代职业教育体系建设，支持湖北科技学院、咸宁职业技术学院提升发展水平。加大教师交流力度，普及教育信息技术，全面推进素质教育。繁荣发展文化事业，提升文化软实力。加强精神文明建设和思想道德建设，挖掘新时期咸宁精神，激发咸宁人热爱咸宁、建设咸宁的主人翁意识。加快推进市传媒大厦、市档案馆图书馆、市妇儿活动中心、市科技馆、市老年人活动中心建设，创建国家公共文化服务体系示范区。深入开展文化惠民工程，丰富群众文化活动，促进公共文化资源和服务体系向基层延伸。深化文化体制改革，推动政府办文化向管文化转变，激发文化创造活力。深入推进医药卫生体制改革，加快新农合市级统筹。继续抓好村卫生室标准化建设，重视基层全科医疗人才培养。加强疾病预防控制和卫生应急服务体系建设，提高公共卫生保障能力。落实国家、省“单独二孩”生育政策，综合治理计划生育率偏低、人口性别比偏高问题。健全养老服务体系，让老年人老有所养老有所乐。加快公共体育设施建设，广泛开展全民健身运动。提高全民科学素养。配合抓好第三次全国经济普查工作。切实保障妇女、儿童、未成年人合法权益。协调发展民族、宗教、外事、侨务、对台、保密、档案、方志、防灾减灾、对口援建等各项社会事业。

提高社会保障水平。落实更加积极的就业政策，坚持以产业促进就业、以创业带动就业，千方百计增加就业岗位。突出抓好高校毕业生、农村转移劳动力、就业困难人员和退役军人等重点人群就业工作。深入推进香城创业计划，加快创建国家级创业型城市。加快完善社会保障制度，进一步扩大社会保险覆盖面，妥善解决被征地农民参加社会保险问题，推进社保体系建设从“制度全覆盖”到“人口全覆盖”。加快保障性住房建设，改善城乡居民居住条件。切实保障低收入群体基本生活，努力实现城乡低保动态管理下的应保尽保，完善社会救助和保障标准与物价上涨挂钩联动机制，探索设立大病医疗慈善救助基金，让困难群众感受到党和政府的温暖。加强“米袋子”、“菜篮子”工程建设，保持物价总水平基本稳定。深入开展放心消费示范景区、放心粮油店、便民餐饮示范店、放心药店、价格诚信店等创建活动，维护消费者权益。

提高社会治理水平。学习“枫桥经验”，创新群众工作方法。加强基层民主政治建设，促进群众在城乡社区治理、基层公共事务和公益事业中依法自我管理、自我服务、自我教育、自我监督。完善基层信访工作网络，畅通行政复议渠道，实行网上受理信访制度，推行“阳光信访”。创新流动人口、特殊人群服务管理，推行流动人口“居住证”制度。牢固树立安全发展理念，全面加强安全生产、消防安全、交通安全、校车安全和食品药品安全。深化“平安咸宁”创建，加强社会管理综合治理，构建立体化社会治安防控体系，依法严厉打击各类违法犯罪活动，让人民群众安居乐业。加强国防动员、国家安全、民兵预备役和人防工作，推动军民融合式发展。

扎实办好惠民实事。今年，市政府继续为城乡居民重点办好“十件实事”：（1）提高就业服务能力，新增城镇就业4万人，扶助创业0.4万人，带动就业1.2万人；实现社会保障卡持卡人数100万，城镇居民省内异地就医联网结算。（2）建成保障性住房10000套（户），新增公共租赁住房7600套，实施棚户区改造15700套，新增发放廉租住房租赁补贴1450户。（3）加强淦河上游水源地保护；解决6万农村人口的饮水安全问题。（4）实施“菜篮子”工程，新建蔬菜批发市场，建设10000亩保障性蔬菜基地，在市中心城区建设10家平价商店，在社区和农贸市场建立50家蔬菜直销店。（5）加强放心粮油市场体系建设，在全市建设6个放心粮油配送中心和50家放心粮油连锁店。（6）加大公交惠民力度，调整优化公交线路，扩大公交覆盖面。（7）深入推进义务教育均衡发展，推动学校标准化、教育信息化建设和师资交流，让乡村孩子享受优质教育资源；在市城区启动新建1所优质公办幼儿园。（8）加强市城区120急救体系建设，规划建设医疗急救站。（9）加强易肇事肇祸精神病人集中救治工作；完成市精神病门诊综合大楼及旧楼改造。（10）推进平安建设，在主城区建设4个警务综合服务站，新建1500个视频探头。

做好今年的各项工作，推进省级战略咸宁实施干在实处、走在前列，人民群众寄予厚望，政府肩负重任。面对新形势、新任务、新要求，必须切实加强政府自身建设，让政府更有效率、群众更加满意。建设学习型政府。加强新理论、新知识的学习，加强新产业、新技术的研究，用创新的思路和办法推动工作。坚持学以致用，不断提高驾驭市场经济的能力，增强应对复杂局面和解决实际问题的本领。推进新型智库建设。建设服务型政府。深入落实中央八项规定、省委六条意见和市委七项要求，扎实开展第二批党的群众路线教育实践活动，坚决反对形式主义、官僚主义、享乐主义和奢靡之风。进一步改进会风文风，减少会议和文件。以钉钉子的精神狠抓落实，推进各项工作具体化、责任化、目标化。坚持问政于民、问需于民、问计于民，健全密切联系群众制度。加大政府购买基本公共服务力度，多做雪中送炭、打基础利长远的实事。深入开展电视问政、行风评议等活动，推进转变作风优化环境集中整治活动常态化。建设法治政府。全面贯彻落实依法治市基本方略，推进法治咸宁、法治政府、法治社会一体建设。自觉接受人大、政协监督，虚心接受社会监督，认真办理人大代表建议和政协委员提案。健全行政决策规则，规范决策程序。加强重大决策全程跟踪反馈和责任追究。完善行政执法体制机制，加强行政执法监督，做到严格规范公正文明执法。在全市各级政府及其工作部门实行权力清单制度，完善政务和各领域办事公开制度，推进决策公开、管理公开、服务公开、结果公开。建设廉洁政府。深入落实党风廉政建设责任制，大力推进惩治和预防腐败体系建设。加强行政监察和审计成果运用，切实把廉政建设的要求落实到政府工作各个环节。严格执行《党政机关厉行节约反对浪费条例》，严格控制“三公”经费支出，政府自觉过紧日子，让人民群众过上好日子！

各位代表！实干成就梦想，奋斗铸造辉煌。让我们在“中国梦”的激励下，紧密团结在以习近平同志为总书记的党中央周围，紧紧依靠省委、省政府和市委的坚强领导，解放思想，攻坚克难，锐意创新，砥砺奋进，为加快推进省级战略咸宁实施干在实处、走在前列而努力奋斗！
